# The role of orienting in vibrissal touch sensing

**DOI:** 10.3389/fnbeh.2012.00039

**Published:** 2012-07-09

**Authors:** Robyn A. Grant, Anna L. Sperber, Tony J. Prescott

**Affiliations:** Department of Psychology, University of SheffieldSheffield, UK

**Keywords:** orienting, whisking, huddling, tactile fovea, development, active sensing, superior colliculus

## Abstract

Rodents, such as rats and mice, are strongly tactile animals who explore the environment with their long mobile facial whiskers, or macrovibrissae, and orient to explore objects further with their shorter, more densely packed, microvibrissae. Although whisker motion (whisking) has been extensively studied, less is known about how rodents orient their vibrissal system to investigate unexpected stimuli. We describe two studies that address this question. In the first we seek to characterize how adult rats orient toward unexpected macrovibrissal contacts with objects and examine the microvibrissal exploration behavior following such contacts. We show that rats orient to the nearest macrovibrissal contact on an unexpected object, progressively homing in on the nearest contact point on the object in each subsequent whisk. Following contact, rats “dab” against the object with their microvibrissae at an average rate of approximately 8 Hz, which suggests synchronization of microvibrissal dabbing with macrovibrissal motion, and an amplitude of 5 mm. In study two, we examine the role of orienting to tactile contacts in developing rat pups for maintaining aggregations (huddles). We show that young pups are able to orient to contacts with nearby conspecifics before their eyes open implying an important role for the macrovibrissae, which are present from birth, in maintaining contact with conspecifics. Overall, these data suggest that orienting to tactile cues, detected by the vibrissal system, plays a crucial role throughout the life of a rat.

## Introduction

Orienting behaviors are a generic aspect of animal sensing; for example, in vision (Land et al., 1999), hearing (Heffner, 1997), echolocation (Valentine et al., 2002), and touch (Catania and Kaas, 1997). Orienting can be defined as bringing your sensory apparatus to a point of interest. In describing orienting as an investigatory reflex, Pavlov (1927) made the following comment:

As another example of a reflex which is very much neglected we may refer to what may be called the investigatory reflex. I call it the ‘What-is-it?’ reflex. It is this reflex which brings about the immediate response in man and animals to the slightest changes in the world around them, so that they immediately orientate their appropriate receptor organ in accordance with the perceptible quality in the agent bringing about the change, making full investigation of it.

(p. 140)

What is interesting here is that Pavlov proposes that it is not just the orient that is important, but also the subsequent exploration movements that contribute to the effective investigation of a stimulus.

Rodents, such as rats and mice, are strongly tactile animals who scan the environment with their long facial whiskers, or *macrovibrissae*, and orient to explore objects further with their shorter, and more densely packed, *microvibrissae* (Brecht et al., 1997; Hartmann, 2001; Grant et al., 2009). Rats have been found to modify their macrovibrissal movements in response to contact, in a manner that appears to regulate the force, number and durations of whisker-surface contacts (Carvell and Simons, 1990; Mitchinson et al., 2007; Grant et al., 2009); vibrissal tactile sensing is, therefore, often seen as a paradigmatic example of an active sensing system (Prescott et al., 2011). Observations from as early as Vincent (1912) and Welker (1964) have described rats “nosing” an object, or directing their nose toward an object, but the orienting movements have yet to be fully described; hence in this article we will seek to better characterize both orienting movement to vibrissal touch, and aspects of subsequent exploration movements, in both adult and neonatal rats, in order to examine some hypotheses about the role of orienting in active vibrissal touch.

### Orienting in vibrissal touch

**Figure 1:**
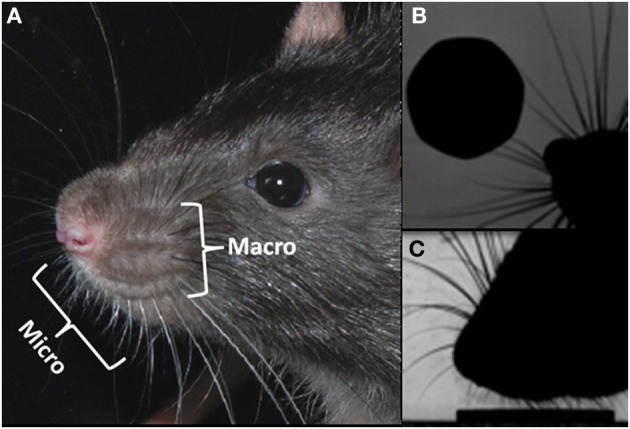
**Macro and microvibrissae. (A)** Photograph of a rats head, showing the micro and macrovibrissa of the mystacial pad; **(B)** overhead view of the rat locating a coin with its acutely protracted rostral whiskers, **(C)** highly zoomed side-on view of the rat “dabbing” its microvibrissae upon the coin, once it has been located.

Vibrissae, in rodents, are the prominent hairs positioned on the upper lip, mystacial pad, lower lip, brow, cheeks, and forelegs (Ahl, 1986). It is the mystacial vibrissae that have excited the most interest, in particular, the tapered macrovibrissae, which are independently moveable vibrissae via intrinsic musculature (Figure 1A). The macrovibrissae are not to be confused with the microvibrissae, which are much shorter (<7 mm) and not independently mobile (Figure 1A). The microvibrissae are also much more numerous—Brecht et al. (1997) counted 90–140 whiskers—and are not solely found on the mystacial pad, but also on the lower jaw and on the inside of the upper lip on the furry buccal pad (Welker, 1964; Brecht et al., 1997). When freely exploring animals contact an object with their macrovibrissae (Figure 1B), they tend to position the microvibrissal region on the items they are investigating (Figure 1C; see also Brecht et al., 1997; Hartmann, 2001). Indeed, the macro and microvibrissae are often employed together in tasks to discriminate between different textures and shapes (Brecht et al., 1997; Hartmann, 2001), and it has been suggested that the macrovibrissae sample spatially in order to orient the microvibrissae. The average whisker density in the microvibrissae array (87/cm^2^) is considerably higher than that of the macrovibrissae (2/cm^2^; Brecht et al., 1997) consistent with this notion that the microvibrissae provide a high resolution sampling area and acting as a tactile “fovea.” The position and small size of the microvibrissae have caused difficulties in studying them, and their use is, therefore, poorly characterized relative to that of the macrovibrissae. Methodological limitations may, therefore, be leading to an underemphasize, in the experimental literature, on the importance of the microvibrissae in rodent tactile sensing.

**Figure 2:**
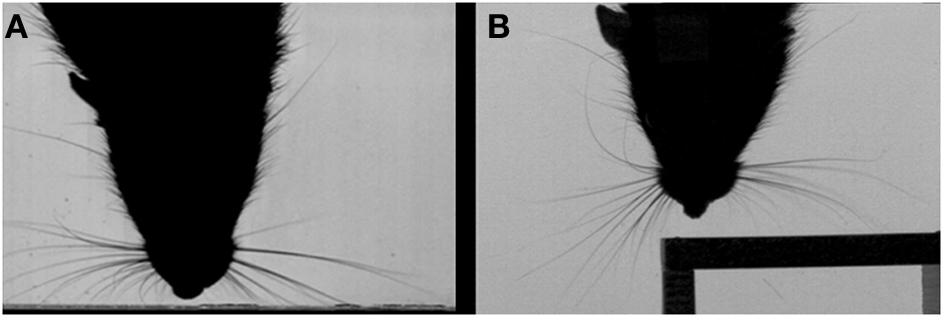
**Contact-related macrovibrissal orienting. (A)** Video still showing and example of a reduction in whisker spread following a contact. **(B)** Video still showing contact-related asymmetry on a corner of a perspex cube. Both panels are based on Figure 1 in Grant et al. (2012).

The orienting behavior that rats perform with their macrovibrissae has been proposed to conform to a “maximal contact” active sensing strategy (Mitchinson et al., 2007; Grant et al., 2009), such that following a contact, as many whiskers as possible are positioned on to the surface, so as to extract more information. Several aspects of whisker control contribute to promote the number of whisker contacts such as the reduction in whisker spread following an initial contact (Figure 2A). Mitchinson et al. (2007), found that in addition to controlling the whiskers so as to increase contact, the movements of the vibrissae may also be regulated so as to “minimize impingement”—the amount of bending of the whiskers against the contacted surface. Hence, when a rat contacts the corner of a perspex block, the macrovibrissae appear to orient with respect to the surface, according to a combined “minimal impingement/maximal contact” control strategy, such that the whiskers ipsilateral to the corner gently touch the surface, whilst those contralateral to the surface “reach round” so as to make additional contacts (Figure 2B) (Mitchinson et al., 2011). In this way the macrovibrissae almost take on the shape of the object in order to better extract certain properties from it. The macrovibrissae can also be oriented ahead of a head rotation, which also gives rise to asymmetry of the whisker fields (Towal and Hartmann, 2006).

In the remainder of this article we describe two studies intended to improve our understanding of the role of orienting in vibrissal touch sensing. In study one, we seek to characterize how adult rats orient toward unexpected contacts on their long vibrissae. We follow Brecht et al. (1997), in assuming that rats will seek to explore a novel object with both their macro- and micro-vibrissae, and that the microvibrissae on the chin and lips can be considered to form a central foveal zone. Orienting, therefore, should consist of moving this fovea to locations of interest on detected objects, then exploring at these locations using motions that appropriately stimulate the microvibrissae. We look both at how macrovibrissal contacts are used to select the initial location for microvibrissal placement, and at how, having oriented, the animals move their short vibrissae to explore across the target surface. In study two, we examine the role of orienting to vibrissal contacts in developing rat pups for maintaining aggregations (huddles). Close contact with conspecifics is critical for the survival of young rat pups in order to maintain body temperature and gain access to food. Orienting at this age, therefore, requires successfully detecting the presence of nearby conspecifics and effectively judging in which direction to move in order to maintain or increase contact. Results of both studies provide new evidence that orienting to tactile cues, detected by the vibrissal system, plays a critical role throughout the life of a rat.

## Study 1: orienting and dabbing in adult rats

In this study we seek to characterize how adult rats orient toward unexpected macrovibrissal contacts with objects, we also examine the microvibrissal exploration behavior following such contacts. Data was recorded in two different settings, one to encourage orienting to a vertical block, the other to encourage exploration through palpation or “dabbing” of a horizontal surface with the microvibrissae. Henceforth these will be referred to as the “orienting” and “dabbing” data-sets.

### Materials and methods

#### Animals

Data was collected using seven male Royal College of Surgeons (RCS) rats, aged 9–13 months and weighing 334–377 g. All animals had genetic retinal degeneration (dystrophy). These animals exhibit normal whisking behavior but also almost completely blind and rely solely on tactile stimuli from their whiskers during testing (Hetherington et al., 2000). Hetherington et al. (2000) found that there was a tactile deficit in an RCS rat's ability to orient to a stimulus on their flank. However, they also tested their ability to orient to a stimulus in the whisker field and found no difference between RCS rats and other strains. In addition, quantification of whisker movements in our laboratory of dystrophic and non-dystrophic RCS animals and of sighted Hooded Lister rats suggest that whisking control in dystrophic animals does not deviate in any marked way from that of normally sighted rats. Therefore, although rats usually use their whiskers in the context of vision, there is no evidence that RCS rats have any deficit in tactile acuity of the whisker system, or use their whiskers in a different way. The rats were kept at 22°C in a 12-h light/dark cycle and had unrestricted access to food and water. All procedures were approved by the local Ethics Committee and UK Home Office, under the terms of the UK Animals (Scientific Procedures) Act of 1986.

#### Procedures

Data collection took place in a 40 × 40 cm rectangular viewing arena with a glass floor, ceiling and front wall. It was illuminated from below by a custom-built high-power light box. Digital high-speed video recordings were made with a Photron Fastcam PCI at 500 frames per second with a shutter speed of 0.5 ms, f-stop 22 and a resolution of 1024 × 1024 pixels. The camera was installed above the arena and thus produced an overhead view. To provide the camera with a second viewpoint, a front-silvered mirror was placed behind the glass front wall, suitably angled to offer a side-on view (as per Grant et al., 2009). Before each session the camera was positioned so that it looked straight down the front wall in the overhead view and the mirror was set at an angle of approximately 45° so that the camera looked along the floor in the side-on view. After precisely positioning the camera, a calibration tool with known dimensions was placed in the arena and recorded to serve as a reference for converting pixels to mm. One of two rectangular cuboids (*blocks*) made of transparent acrylic glass was placed in the arena and affixed to the floor with blue tack.

**Figure 3:**
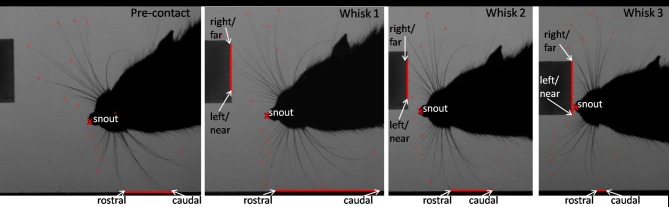
**An example of the tracked points measured during orienting.** Example video stills from pre-contact whisk to a microvibrissal contact in whisk 3. Whisk 1: the rat has just contacted the block. Second whisk: the rat orients toward the block. Third whisk and microvibrissal contact: the nose is now touching the block. The red lines correspond to the area of whiskers contacting the wall and perspex block. The rat moves away from the wall and toward the novel perspex block. The snout position and the positions of the whiskers on the block (right, left, near, and far), and the wall (rostral and caudal) will be to construct our measures, which we describe below.

**Figure 4:**
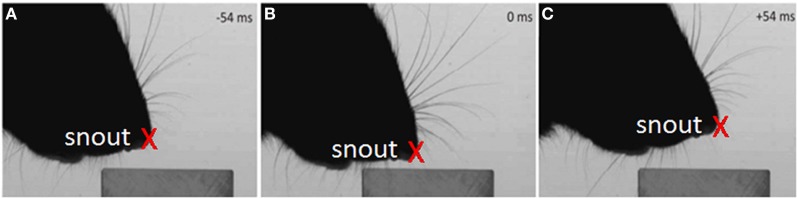
**Example of the tracked nose during the dabbing clips, shown in three example video-stills**.

For recording of the orienting data-set a block with 2.0 × 2.8 × 7.65 cm edge length and a 50 mm lens were employed (as in Figure 3). To observe “dabbing” the block had the dimensions of 2.45 × 3.0 × 1.35 cm and a 120 mm lens was used (as in Figure 4). While the taller block offered a bigger area for the orienting behavior, the smaller block size provided a surface at a suitable height to encourage microvibrissal exploration.

#### Recording

Data was collected in daily sessions in which each rat was individually placed in the viewing arena and allowed to freely explore its surroundings. During exploration, recordings of 1.6 s length were taken opportunistically when the rat contacted one of the perspex blocks. Only one perspex block was used in a recording session, and we alternated between the orienting block and the dabbing block on a daily basis. Generally, 12 clips were recorded for each animal per session. When a rat showed no exploratory behavior for more than 5 min, however, recording of that animal was discontinued for that session. A total of 129 clips were recorded for the orienting behavior (15–25 per animal) and 172 for the dabbing behavior (13–35 per animal).

#### Data selection

Clips were selected for further analysis as follows. By manually inspecting the videos, frame by frame, the times (frames) of maximum macrovibrissal protraction were identified separately for the right and left whisker arrays this allowed each clip to be decomposed as a series of whisk cycles (“whisks”). Microvibrissal contact in both the orienting data-set and the dabbing data-set was inferred when the snout tip touched the block, which could generally be discerned in the side-on camera view.

For the orienting data-set the clips were required to have an initial whisk cycle in which there was no whisker contact with the block, termed a *pre-contact whisk*, to ensure that the first macrovibrissal contact with the block was always recorded. Each clip also had to provide a clear view of the rat making contact with the object, first with the macrovibrissae and subsequently with its microvibrissae. Eighty-four clips were discarded, most of them because the pre-contact whisk was missing (28 clips). In 18 clips there was no orientation or microvibrissae contact and in 10 clips the animal's snout was obscured by the block in the side-on view. In total, 45 clips (2–11 per animal) in the orienting data-set match the criteria for further analysis. In these clips the initial microvibrissal contact occurred in either the 3rd or 4th whisk following the initial pre-contact whisk, therefore clips were analyzed for either 3 (12 clips) or 4 (9 clips) consecutive whisk cycles.

For the dabbing data-set the clips were required to include at least one complete episode in which the tip of the animal's snout approached the block, made microvibrissal contact, and then withdrew—defined as a “dab.” The snout tip was required to be visible at all times. One Hundred clips were rejected, due to the snout being obscured (59 clips) or the rat showing no dabbing behavior (18 clips), this left a total of 72 (4–15 per animal) clips for further analysis in the dabbing data-set.

#### Data analysis

Selected clips were examined by eye on an LCD monitor using uncompressed video and a purpose-built whisker tracking/analysis tool (as used by Mitchinson et al., 2007; Grant et al., 2009). In each clip, for both the orienting and dabbing data-sets, the corners of the block were identified in both the overhead and the side-on view to provide accurate information about the block's position.

#### Analysis of the orienting data-set

Each clip can be decomposed into a sequence of whisks. We refer to the ith whisk in the sequence where *i* ∈ {0, 1, 2, 3, 4} whisk 0 is the pre-contact whisk and the snout contacts the block in either whisk 3 or whisk 4. We focus on the frames in which the whiskers are identified to be at maximum protraction, an example of which is shown in Figure 3. The vector position of the snout tip (s) and of the locations of key whisker-object contact locations are determined by visual inspection and marked as shown in the example frames (Figure 3). For each sequence these locations are then used to define the following measures:

s_*Final*_: The position of the snout tip in the *i*th whisk and in the *final* whisk, the latter defines the point of microvibrissal contact.
*p*^*bk*^_*i*_, *p*^*wk*^_*i*_: A point *k* on the block (*b*) or wall (*w*) identified in an image frame corresponding to the *i*th whisk.
*p*^*bLeft*^_*i*_, *p*^*bRight*^_*i*_, *p*^*bNear*^_*i*_, *p*^*bFar*^_*i*_, *p*^*wRost*^_*i*_, *p*^*wCaud*^_*i*_: Respectively, the positions of the left-most and right-most macrovibrissal contacts points on the block, the nearest and furthest macrovibrissal block contact points, and the most rostral and most caudal contact points on the wall; all as determined by visual inspection of the relevant frames for whisk *i*. Note that, since the block is to the right of the wall, the leftmost and nearest contacts points are often the same, and the rightmost and furthest contact points are often the same.
*p*^*bMid*^_*i*_, *p*^*wMid*^_*i*_: The mid-point of contacts with the block (*b*) or wall (*w*) calculated as the vector average of the left-most and right-most contacts points on the block and of the most rostral and most caudal contact points on the wall, i.e., $p_{i}^{bMid}=\frac{1}{2}\sum_{k}\left(p_{i}^{bLeft}+p_{i}^{bRight}\right)$, $p_{i}^{wMid}=\frac{1}{2}\left(p_{i}^{wRost}+p_{i}^{wCaud}\right)$.
*d*^*bk*^_*i*_, *d*^*wk*^_*i*_: The distance from the snout tip to a given point on the block or wall, e.g., *d*^*bNear*^_*i*_ = ║s_*i*_ −c^*bNear*^_*i*_║, *d*^*wMid*^_*i*_ = ║s_*i*_ −c^*wMid*^_*i*_║.
*d*^*bk*^_*ij*_: We wish to be able to compare the snout position in whisk *i*, with whisker contact position in a previous whisk cycle *j*, we, therefore, define *d*^*bk*^_*ij*_ = ║s_*i*_ −c^*bk*^_*j*_║. For instance *d*^*bNear*^_32_ = ║s_3_ −c^*bNear*^_2_║ is the distance from the snout position in whisk 3 to the nearest macrovibissal contact position on the block in the preceding whisk 2.

To examine whether animals orient away from the wall and toward the block we perform repeated measures ANOVA for the measures *d*^*bMid*^_*i*_ and *d*^*wMid*^_*i*_ for *i* = 1,…, 4. To examine whether the rat orients toward the nearest, furthest, or intermediate point of contact on the block, and to the position detected in the initial contact (whisk 1) or in the most recent contact (whisk *i*−1) we looked at values of *d*^*bk*^_*i*1_ for the positions *near*, *far*, and *mid*, and *d*^*bk*^_*i*(*i−l)*_ for *l* = 1,…, 3. We also performed a regression of the *y*-coordinate of the final microvibrissal contact position s_*Final*_ with the *y*-coordinate of *p*^*bNear*^_1_, *p*^*bFar*^_1_, *p*^*bMid*^_1_, *p*^*bNear*^_(*Final*−1)_, *p*^*bNear*^_(*Final*−2)_. All data was checked to ensure normal distributions (Kolmogorov–Smirnov Test) and equal variances (Levene's Test) before running the ANOVA and regression analyses.

#### Analysis of the dabbing data-set

The tip of the snout was tracked in every fourth frame in both the side-on view (Figure 4) and the side-on view, with intervening values computed by interpolation. The change in *y* co-ordinate of the snout position over time was then used to detect the “dabbing” motion for which measures of dab amplitude and frequency were calculated as follows.

Dab *frequency* was estimated using an autocorrelogram of the tracked vertical position. Specifically, the time series was smoothed using a zero-phase low-pass filter (boxcar) with a cut-off frequency of 16 Hz, which is well above the highest expected dab frequency from looking at the footage, this removes the high-frequency information. The first peak (maximum) in the autocorrelation of this smoothed time series was then identified automatically to give a first estimate of signal period. This estimate was then refined by gradient ascent on the unsmoothed autocorrelation series to locate the nearest peak to that found automatically in the smoothed.

To estimate the dab *amplitude* we first removed the mean value from the vertical nose position time series then computed the root mean square value to give the root-mean-square (RMS) dabbing amplitude. These time series were approximately sinusoidal, so we were able to estimate the “peak-to-peak dab amplitude” by multiplying the RMS dabbing amplitude by 2v2 (Chatfield, 2003). This estimate of amplitude is reasonably robust to departures from a purely sinusoidal pattern.

### Results

#### Rats orient toward unexpected object contacts

**Figure 5:**
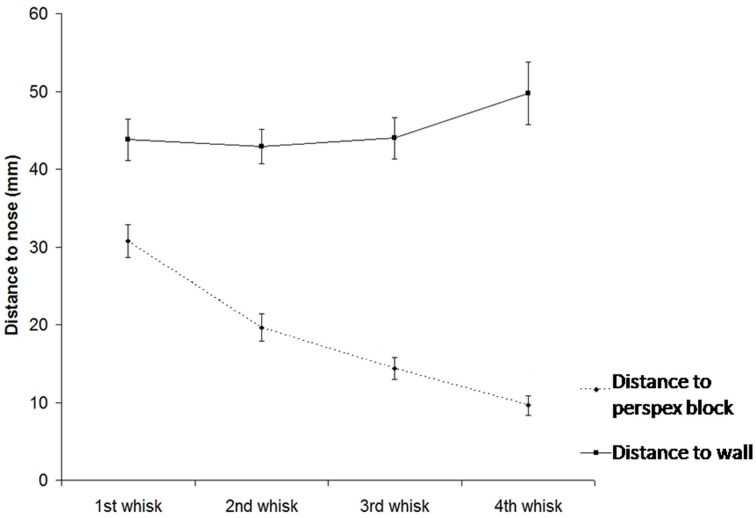
**Position of the snout (S) relative to the mid-point of contacts wall and with the block (as detected by the macrovibrissae) over subsequent whisks.** The graph shows that animals toward the block over time indicating orienting toward the unexpected object, gradually moving away from the wall as they do so.

As show in Figure 5, when animals make an unexpected contact with an object, they will orient toward that object over successive whisk cycles (repeated measures ANOVA for *d*^*bMid*^_*i*_ and *i* = 1,…, 4: [*F*_(3, 33)_ = 54.8, *p* = 0 < 0.001], *post-hoc* tests: *d*^*bMid*^_1_ > d^*bMid*^_2_ > d^*bMid*^_3_ > d^*bMid*^_4_). At the same time they also gradually move away from the wall that was previously being followed (repeated measures ANOVA for *d*^*wMid*^_*i*_ and *i* = 1,…, 4: [*F*_(2, 18)_ = 0.91, *p* = 0.010], *post-hoc* tests: *d*^*wMid*^_1_, d^*wMid*^_2_, d^*wMid*^_3_ <d^*wMid*^_4_). This occurs even when the rat is contacting the wall and the block with whiskers on the same side of the face.

#### Rats orient toward the closest whisker-object contact position

**Figure 6:**
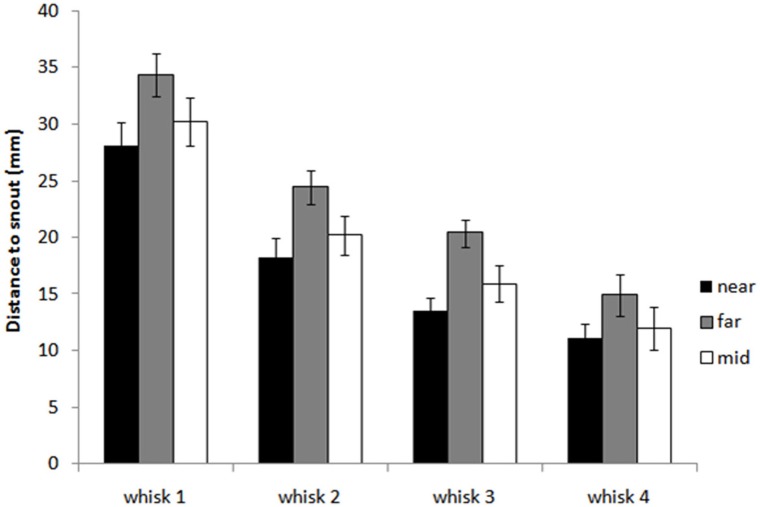
**Distance from the snout tip in each whisk cycle to the nearest, furthest, and mid-point of block contacts in the initial contact whisk.** The graph shows histograms of the distances *d*^*bNear*^_*i*1_, *d*^*bFar*^_*i*1_ and *d*^*bMid*^_*i*1_ for whisks *i* = 1,…, 4, averaged for X clips. The plot shows that as the animal orients toward the block, in whisks 2, 3, and 4, it moves toward the nearest initial point of contact rather than the furthest point or an intermediate point.

Figure 6 shows that animals tend to orient toward the nearest initial whisker-object contact position rather than the furthest initial contact point or the mid-point of the initial contacts. An example of orienting was given in Figure 3 which shows the whiskers positioned at maximum protraction for a sequence of four whisks, with initial macrovibrissal contact shown in the second frame, and microvibrissal (snout) contact in the final frame. Across the series the animal can be seen to move away from the wall and toward the nearest whisker contacts.

**Figure 7:**
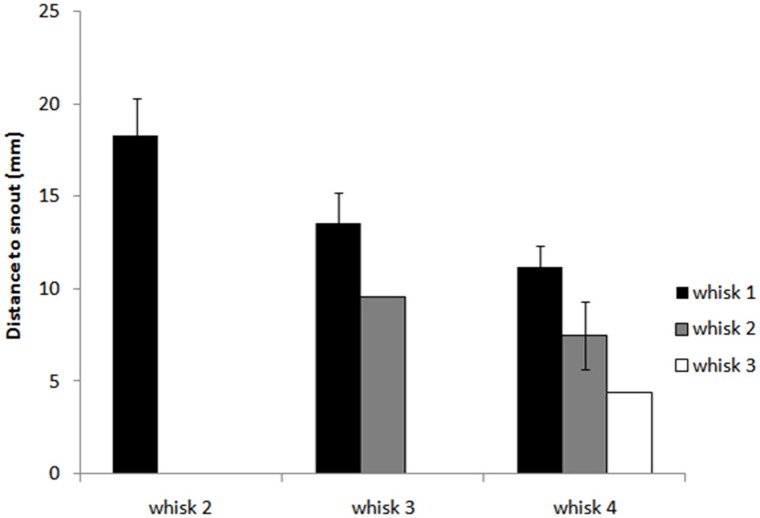
**Distance from the snout tip (s) in each whisk cycle to the nearest point of block contacts in preceding whisk cycles.** The graph shows histograms of the distances *d*^*bNear*^_*i*(*i*−*l*)_ from the snout tip to block contacts for whisks *i* = 1,…, 4 and for *l* = 1,…, 3, averaged for X clips. For whisk 2, *d*^*bNear*^_21_ is shown as a reference (there are no earlier contacts to compare to), for whisk 3 we can compare *d*^*bNear*^_31_ and *d*^*bNear*^_32_, and for whisk 4 *d*^*bNear*^_41_, *d*^*bNear*^_42_, and *d*^*bNear*^_43_. The plot suggest that as the animal orients toward the block it progressively adjust its trajectory to move toward the nearest point of contact in the immediately preceding whisk.

Figure 7 and regression analyses (see Table 1) indicate that the animal may progressively home in on the position of the nearest macrovibrissal contact in preceding whisks. This result should be taken with some caution, however, since the most recent contact could be a better predictor simply because it is more proximal in time. Confirmation that the trajectory was been updated/corrected on a per-whisk basis could be found if the head position could be observed to fluctuate on the same time-scale as the whisk cycles. Informal observations of the whisker and head movements in all the 59 tracked orient examples did not identify any clear examples of such correctional movements. However, even in the absence of such evidence, per-whisk corrections could be taking place, these might either be too small to be apparent or may be obscured by some smoothing process that affects the orienting behavior.

**Table 1:** **Regression analyses for the *y*-coordinate of the microvibrissal contact position (s_*Final*_) with the *y*-coordinate of vibrissal contact positions in the first and subsequent contact whisks**.

	***p*^*bNear*^_1_**	***p*^*bFar*^_1_**	***p*^*bMid*^_1_**	***p*^*bNear*^_(*Final*–2)_**	***p*^*bNear*^_(*Final*–1)_**
*R*^2^	0.758	0.522	0.673	0.886	0.901

A linear regression model of the *y*-coordinate of the final microvibrissal contact position s_Final_ with the y-coordinate of p^bNear^_1_, p^bFar^_1_, p^bMid^_1_, p^bNear^_(Final–1_), p^bNear^_(Final-2)_ shows that the microvibrissal contact may occur toward the closest whisker contact of preceding whisks and the best predictors are the contact points nearest to the snout tip.

#### Dabbing occurs at a behaviorally relevant frequency

**Figure 8:**
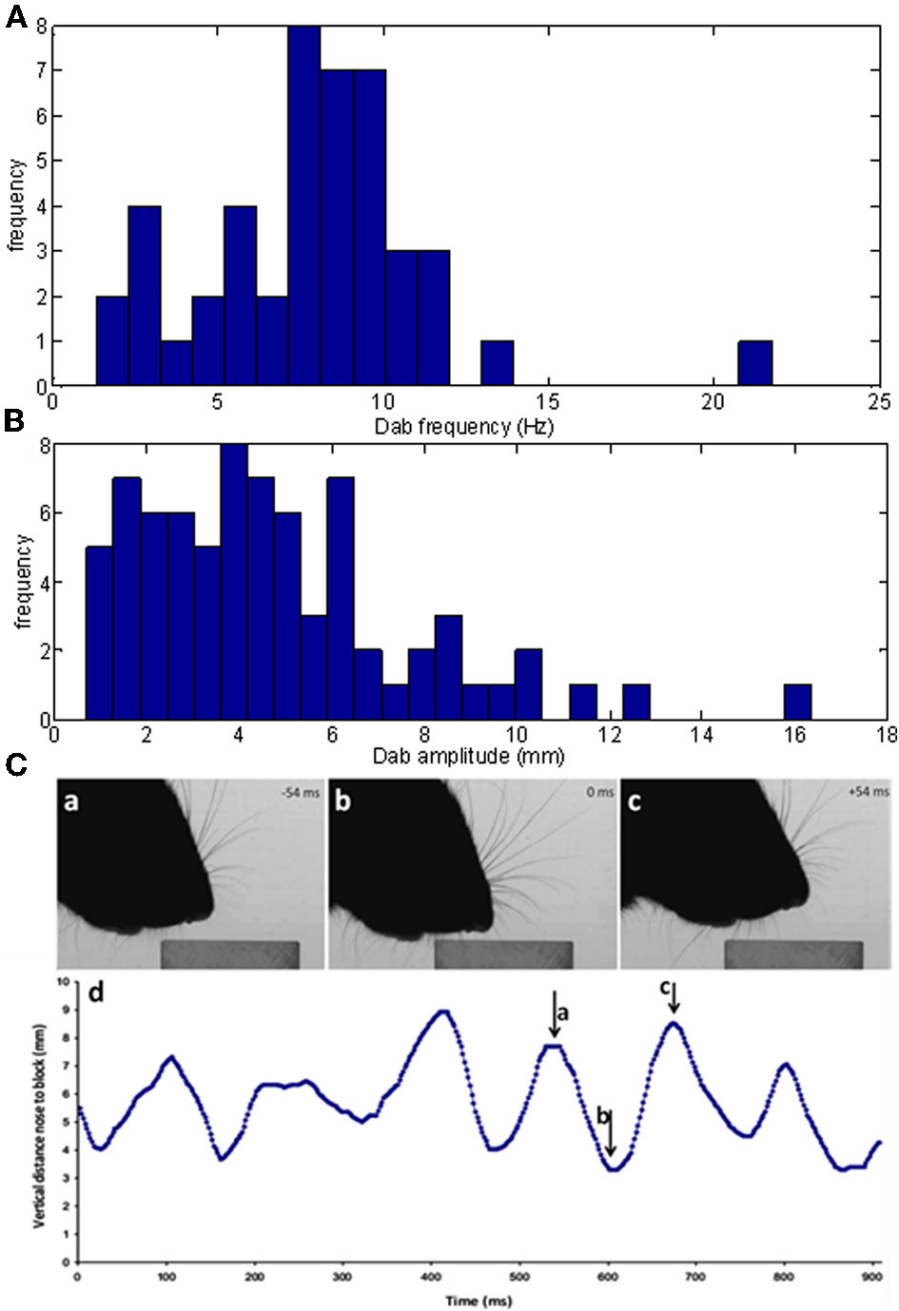
**Characteristics of vertical dabbing movements. (A,B)** Distribution of dabbing frequencies and amplitudes. The mean frequency is at 7.83 Hz and mean amplitude at 43.27 mms. **(C)** Example dabbing motion. The vertical distance, in mm, of the snout tip to the surface of the block in the side-on view plotted against time. Pointers a–c refers to the corresponding screenshots in Figure 4 which used video stills from the same sequence.

If head movements during an orient are relatively smooth, once the microvibrissae touch the block, head movements become very different. In the case of contact with a horizontal surface, the head moves up and down in a rhythmic movement referred to as palpating or dabbing. As shown in Figure 8 the analysis of our dabbing data-set found that these movements occur at around 8 Hz (7.8 ± 3.3 Hz) and at amplitudes of around 5 mm (4.7 ± 3.1). Dabbing is thus on a behaviorally relevant timescale compared to the movements of the macrovibrissae which likewise occurs at a frequency of around 8 Hz (Mitchinson et al., 2011).

For example, Figure 8C shows the vertical position of the snout tip throughout an example video clip; panels **A–C** show example video stills corresponding to the arrows on the graph. We can see here that the snout tip is moving in a rhythmic manner. The video stills show that when the snout tip is away from the block (panels **A** and **C**), the macrovibrissae are retracted and lifted up, but when the head is brought down to palpate the block, the macrovibrissae are protracted and moved downwards to also touch the block. Between dabs the microvibrissae do not always lose contact with the object but rather may be moved along the surface (Figure 8C), suggesting that dabbing does not necessarily create absolutely discrete tactile impressions.

**Figure 9:**
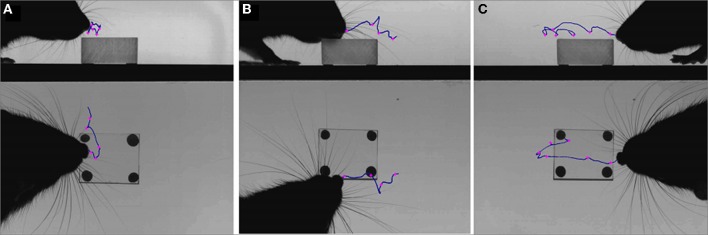
**Dabbing trajectories and microvibrissae contacts.** High-speed video frames from three different clips **(A–C)** overlaid with the trajectory of the nose (blue lines) and the microvibrissae contacts (pink crosses) during a bout of dabbing.

Figure 9 shows three further example dabbing sequences. When dabbing over a block the microvibrissae are moved across the surface, and can sample block edges (Figure 9B) and surfaces (Figures 9A,C). Informally we have observed that, following an orient, the rat may make just the one dab or, if it appears to be interested in actively exploring the object, this is followed by a variable number of others (we have seen up to eight dabs). The number of dabs and the duration of this exploratory behavior is likely to be dependent on both the alertness and motivation of the rat, and the novelty of the stimuli, the effects of such variables could be investigated in future research.

## Study 2: orienting in development

In study two, we examine the role of orienting to vibrissal contacts in developing rat pups for maintaining aggregations (huddles), a behavior that is critical for effective thermoregulation in juvenile animals. Behavior was recorded in two settings. First, we filmed rat pups in their home-cage, using a normal speed camera, interacting with and orienting to conspecifics. As is typical for this type of study quantification of behavior was performed by trained observers using standardized ordinal scales (Sullivan et al., 2003; Grant et al., 2012). To further examine orienting movements in relation to contacts, we also made some high-speed video recording of pup behavior in a setup that was intended to approximate conditions in the home-cage whilst allowing appropriate back-lighting for high-speed videography.

### Materials and methods

#### Animals

Four litters were used in this study, two dystrophic RCS litters and two Hooded Lister litters. The RCS animals have a specific mutation that causes a gradual degeneration of the retina, with the consequence of loss of vision in the mature animal at around 17 weeks of age (Hetherington et al., 2000; McGill et al., 2004), but are normal at the ages tested here (i.e., the rats are able to see as soon as their eyes open and throughout this study). In the adult RCS rat there is no deficit in their ability to orient to a stimulus in the whisker field (Hetherington et al., 2000). Using these two strains gives us a wider distribution of litter sizes, from 6 to 11 animals, as the RCS animals tend to have smaller litters (6 and 8 pups per litter in this study) than the Hooded Listers (7 and 11 pups per litter in this study). Animals were kept in their home cage with both their parents and all their litter-mates present throughout experimentation. The pups were on a 12:12 light schedule and kept at 22°C, with water and food *ad libitum*. All procedures were approved by the local Ethics Committee and UK Home Office, under the terms of the UK Animals (Scientific Procedures) Act, 1986.

#### Procedures

This study employed a form of *focal sampling*, where an individual is scored for a catalogue of behaviors over a limited time period (Altmann, 1974; Martin and Bateson, 1993). Specifically, two individual focal pups were selected at random, one male and one female—eight in total, from each litter and identified from their litter-mates with a marker pen bar on their tail. Animals were filmed between P2 and P21, every day where possible (where the animals are born on P0 and P1 is the subsequent day). The pups were filmed at 25fps using a *Casio Exilim* camera in their home cage in the lab for approximately 8 min per litter. This took place at around 9.30 a.m. each day, with the lid of the cage removed for clear viewing. Sixty-six clips were collected in this way. 9.30 a.m. was selected as a good time of day as there were minimum disturbances to the animal houses in the morning, in addition, informal observations suggested that the animals were more active in the mornings.

Two focal pups in each of the two RCS litters, one male and one female, were also filmed using high-speed videography at P5 and P6. Previous studies have indicated how important realistic temperatures and textures are in eliciting huddling behaviors (Campbell and Raskin, 2004). In order for the focal pup to be clearly visible in the camera view, we therefore put the rest of the litter in a soft material net, with holes of around 4 mm. This meant that the focal pup was in contact with its littermates directly through the holes and would receive tactile cues similar to those obtained in the nest. The netted huddle was placed into a glass experimental arena and the focal pup was positioned to the right of the huddle, approximately 50 mm away. The recording setup and camera were otherwise similar to that used in the high-speed videography element of study one. 1.6 s clips were recorded manually using a trigger whenever the focal pup contacted the netted huddle, an example clip can be seen in Figure 13. When the focal pup moved out of the field of view, it was placed back in to the starting position. Clips were collected in this way to qualitatively illustrate huddling behaviors.

#### Data analysis

**Figure 10:**
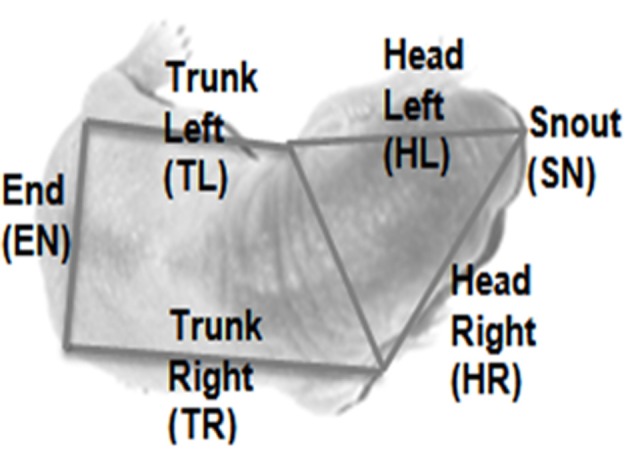
**Body zones for scoring contact type.** The image shows a typical overhead view of a rat pup overlaid with the zones in which the body is segmented in order to assess contact type.

A 40 s sampling time (1000 frames) was selected to provide high sample numbers at a relevant time-scale for the types of behavior (orienting and huddling) we are interested as determined by reviewing pilot footage. At each 40 s interval, each of two focal pups in each litter was given a score on the following measures:

*Huddle size (H1):* the number of animals in the focal pup's huddle or “aggregon” (a group of pups that are in contact with each other), scored between 1 and *n* (the total number of pups in the litter).
*Contact type (H2):* Whereabouts on the body the focal pup it is in contact with its conspecifics, scored either 1 or 0 for six zones as shown in Figure 10.
*Movement direction (H3):* The direction of movement of the focal pup in the subsequent 1 s of footage, scored in one of six possible directions (including no movement).
*Huddling behavior (H4):* The type of huddling behavior engaged in for the next 15 s, scored in one of four categories for the Hooded Lister litters only. Further details of each of the ordinal scales are given in Table 2.

**Table 2:** **Ordinal scales for scoring orienting behavior during rat pup huddling**.

	**Measure**	**Scored**	**Description**
H1	Huddle size	(1−*n*)/*n*	How many pups are in the aggregon (huddle) of the focal pup, normalized to litter size (*n*)
H2	Contact type	Score = 0 or 1 for each of SN, HR, HL, TR, TL, EN	Records where contacts are on a focal pup. Contacts could be trunk left (TL), end (EN), trunk right (TR), head left (HL), head right (HR) and snout (SN) for head contacts that were “straight on.” See Figure 9 for an indication of how these zones are identified in the overhead camera view
H3	Movement direction	Score = 1 for one of None, Left, Right, Forward, Other	The global direction of the focal pups' movements, in the 1 s immediately following the start of the observation period
H4	Subsequent behavior	Score = 1 for one of None, Digging, In-and-around, Away	Records the behavior that follows the contact and initial movement: no movement with respect to huddle position, digging in to the huddle, moving in and around the huddle, moving away from the huddle. Scored for the 15 s immediately following the start of the observation period for the Hooded Lister pups only

Ordinal scales for coding locomotion and huddling, from home cage footage of neonatal rats. All measures except were developed specifically for the current study other than H1 which is taken from Schank and Alberts (1997).

This analysis provided 10–20 data points for each scale, per day, per focal animal.

The principle analysis was to look at the effect of *Contact Type* on the subsequent movement direction of the pup; for instance, which way would the pup move if there was a contact at head-left? For this analysis, a mixed-model ANOVA was carried out with *Contact Type* on the focal pup as a within variable (SN, HL, HR, EN, TL, TR) and *Movement Direction* (none, left, right, forward) and age (immature P2–09, transitory-adult P10–21) as between factors. All the data was checked that it was normally distributed (Kolmogorov–Smirnov Test) and had equal variances (Levene's Test).

### Results

#### Young rat pups turn toward contacts with conspecifics

**Figure 11:**
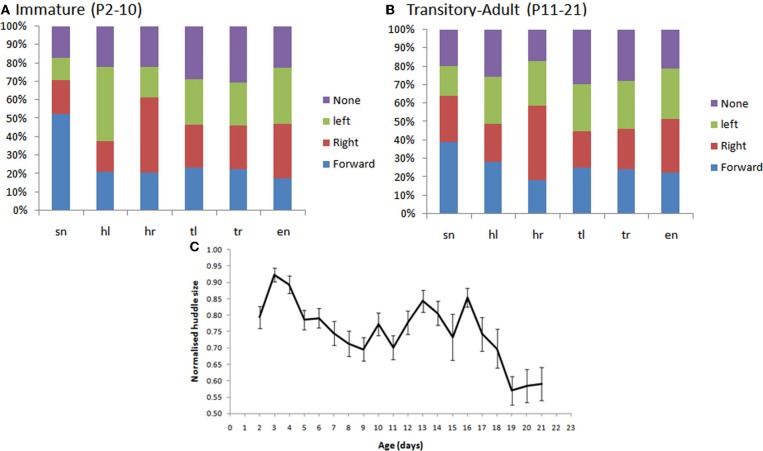
**(A,B)** The percentage of subsequent movements in response to contacts by conspecifics in immature pup, P2–10 (panel **A**) and transitory-adult pups (P11–21). In particular, we see that there are significantly more snout contacts when the rat moves forward, in immature animals, than in transitory-adult pups. [*F*_(3, 79)_ = 9.121, *p* < 0.001; Figure 7B]; more leftward movements following a left head contact [*F*_(3, 79)_ = 6.857, *p* < 0.001], and more rightward movements following a right head contact [*F*_(3, 79)_ = 8.018, *p* < 0.001]. **(C)** The size of the huddle (normalized for litter size) decreases in the third postnatal week (P18+).

As shown in Figure 11A, we found that younger rat pups (P2–10) turn toward conspecifics in their huddle, more frequently than would be expected by chance. Specifically, pups that are contacted on the snout (sn) are more likely to move forward, those contacted on the left of the head (hl) to orient toward the left, and those on the right of the head (hr) toward the right. Older animals (P11–21, Figure 11B) showed no consistent pattern in their responses, possibly because their behavior is becoming too complex to be adequately described by an analysis of the type used here. One reason for the loss of consistency is the general reduction in huddling in older animals. Specifically, and in line with earlier studies (Schank and Alberts, 2000; Schank, 2008), we found that older animals (P18+) spend less time in huddles and were, therefore, less likely to be found in contact with conspecifics (Figure 10C).

#### Rat pups dig in to the huddle, or move in and around it, from P2–10

**Figure 12:**
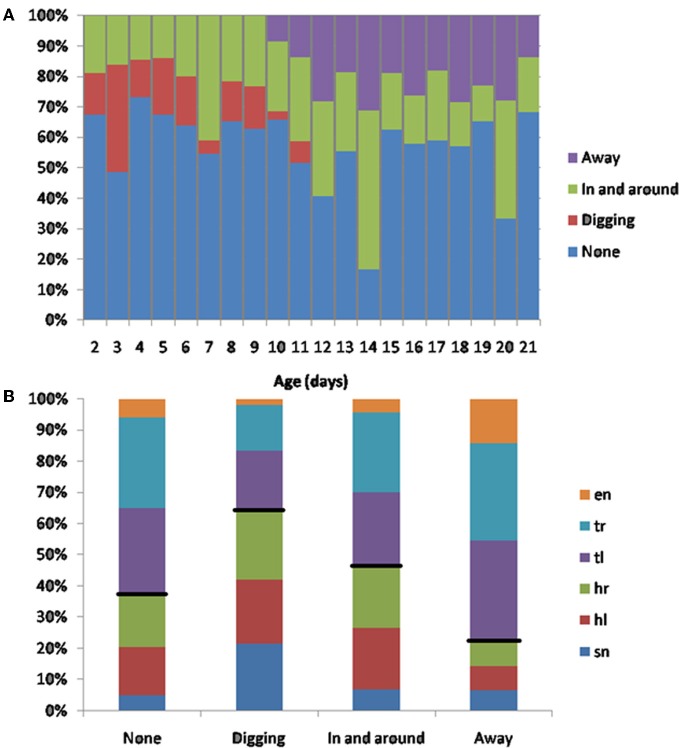
**(A)** Subsequent behaviors following contact. Younger animals (<P12) tend to move in and out of the huddle, or to engage in digging in to the huddle. Older animals spend more time moving away from the huddle (>P10), and no time digging against each other. **(B)** The percentage of contact types for each behavior. Digging tends occur after more snout contacts (indicated by a larger blue bar in the digging category), moving away tends to occur after more trunk contacts (indicated by smaller blue, red and green bars in the away category). Black lines indicate the division between head and trunk contacts.

In a subset of animals (Hooded Lister pups) we also looked at the types of huddling behavior engaged in by animals that were in contact with conspecifics. Specifically, we observed whether the pup moved in-and-around the huddle, buried itself deeper into the huddle, or moved away from the huddle. As shown in Figure 12A, prior to P12, movement with respect to the huddle, where present, tended to involve moving in and around the huddle, or digging-in—burying under other animals in the huddle. At later ages, beginning from P10 pups move away from the huddle in some clips showing the beginnings of exploratory behavior. Figure 12B, shows that the “digging-in” behavior is more frequent when there is a snout contact (Figure 11B), suggesting a possible tactile trigger for this behavior.

**Figure 13:**
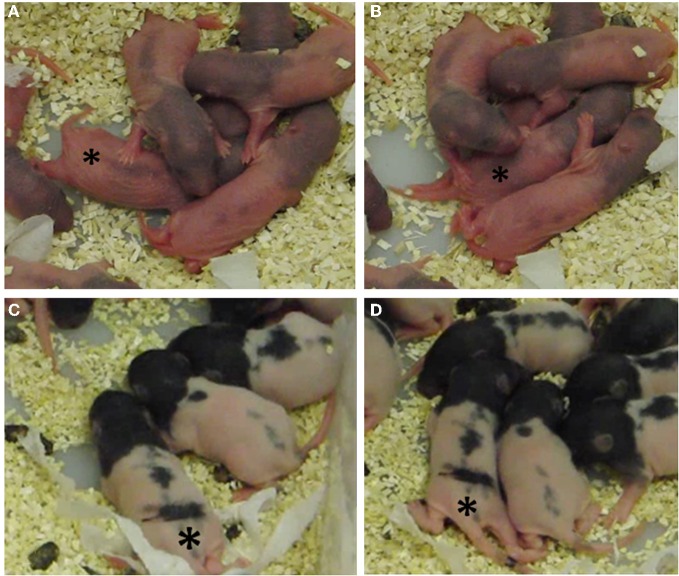
**Video stills of P2 and P7 pups in their home cage. (A)** A P2 focal pup moves forward following contacts on its head left and head right; **(B)** 3.2 seconds later, the pup starts to dig in to the huddle, indicated by the extended rear limbs. **(C)** A P7 focal pup has been contacted on its head right; **(D)** 4.3 seconds later the pup has oriented right toward the contacts and starts to move in and around the huddle. In each still the focal pup is indicated by an asterisk (*).

Examples of huddling can be seen in the video stills in Figure 13, taken from two different filming sessions. Panels **A,B** shows P2 pups in a huddle. The focal pup (indicated by the asterisk) is being contacted by its conspecifics on its head left and head right (Figure 13A). The pup then begins to dig into the huddle, which can be seen by its extended rear legs (Figure 13B). Figure 13C shows a P7 pup being contacted on head right, it turns toward the contact (Figure 13D) and moves further into the huddle.

**Figure 14:**
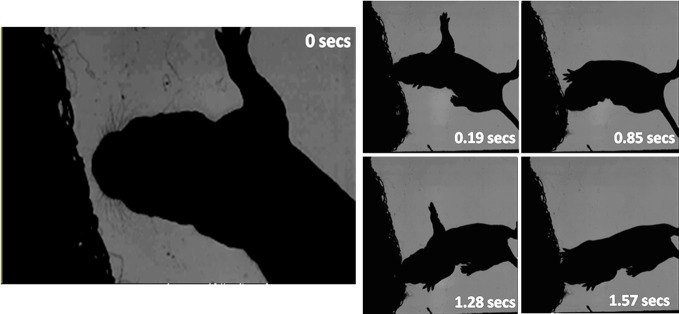
**High-speed frames showing a P6 rat interacting with a netted huddle.** The first whisker contact is at 0 s on head left. The pup continues to move its head forward into the contact, which causes a deeper snout contact (0.19 s). The pup “dabs” its head in to the huddle (0.85 s), positions into a gap (1.28 s) and starts to dig further into the huddle (1.57 s).

The home cage study demonstrates that young pups will orient toward conspecifics at an age before they eyes have opened, however, this does not confirm specifically that the trigger for the orienting movement will have been a vibrissal contact. At this age, the vibrissae are extremely fine hairs and impossible to observe in home cage video footage of this type. To confirm a role for whiskers in orienting we, therefore, examined pup orienting behavior in our high-speed video footage where we filmed pups interacting with a netted huddle of conspecifics. These recordings indicated that vibrissal contacts can serve as a trigger for orienting movements. For example, In Figure 14, a P6 rat pup can be seen making whisker contacts with the netted huddle on the left side of its head (0–0.19 s). It then “noses” around the huddle until the snout is positioned into a gap, and then starts to “dig” into the huddle.

## Discussion

In study one, we showed that rats orient to the nearest macrovibrissal contact on an unexpected object, progressively homing in on the nearest contact point on the object in each subsequent whisk. Although it is not surprising that rats choose to move toward the nearest contact point it is at least logically possible that they might target the average position of vibrissal contacts on the object, therefore it is useful to confirm that the nearest contact point is generally preferred. Targeting the nearest point has two benefits. First the animal will be able to approach that point more quickly than any other point, second, it will expend less energy and be detoured less from its previous path in doing so, than if any other point of contact is targeted. Therefore, the strategy adopted by the animal is clearly an efficient one for rapidly approaching and finding out about novel objects.

The midbrain superior colliculus is widely considered to play a crucial role in orienting toward spatial targets (Benedetti, 1991; Hemelt and Keller, 2007; Cohen et al., 2008). That we are suggesting that the rat might be orienting to closest whisker contacts appears consistent with Cohen et al.'s (2008) findings that the colliculus responds most strongly to early contacts. In our study the closest contacts tended to occur on the more rostral whiskers, which do tend to contact surfaces before the more the caudal whiskers, which make contact with points more distal from the snout tip as they are swept forward. Benedetti (1991) found that whiskers more central to the visual field project to larger areas in the colliculus, again this could potentially also lead to greater preference for rostral whiskers in selecting the target for orienting movements.

Following contact, rats “dab” against the object with their microvibrissae at an average rate of ~8 Hz which is consistent with Hartmann (2001) who showed evidence of the synchronization of microvibrissal dabbing with macrovibrissal motion. Here we have provided additional evidence concerning the amplitude of the dabbing motion which was found to be around 5 mm in our data, though very variable. Further examination of microvibrissal contact patterns would be useful to understand better how animals are using this high-resolution region of the vibrissal sensing system.

In study two, we examined the role of orienting to tactile contacts in developing rat pups for maintaining aggregations (huddles). The successful maintenance of huddling is crucial for the survival of young rat pups, so they can successfully feed and stay warm. Sullivan et al. (2003) previously showed that the loss of vibrissae could impair effective huddling behavior. That vibrissae are already present at birth and can solicit head turning in very young animals (see also Grant et al., 2012) is further evidence that the vibrissae play an important role in maintaining contact with conspecifics. In the current study we have provided evidence consistent with the hypothesis that young pups are able to orient to contacts with nearby littermates before their eyes open. High-speed video footage confirms that contacts on the vibrissae are a likely candidate for the tactile triggers underlying this behavior. Interestingly, the superior colliculus appears to mature relatively slowly in young rat pups not reaching adult-like activity levels until the third week. The mechanisms underlying orienting in young rat pups, therefore, remain to be identified. It might be that the low numbers of unorganized somatosensory units in the colliculus are sufficient to drive these simple behaviors, alternatively brainstem systems outside the colliculus may be adequate to co-ordinate some forms of orienting. In addition, these behaviors seem to be only elicited by realistic whisker contacts, for example rat pups cannot orient toward whisker deflections with a wooden rod until after eye-opening (around P12; Sullivan et al., 2003). Indeed Campbell and Raskin (2004) showed that for a rat pup to show normal huddling behaviors it has to make contact with something warm, soft, and fluffy. These factors are all crucial for a young pup to successfully turn toward conspecifics and their whiskers may play a role in identifying the tactile characteristics of conspecifics as well as locating them in nearby space.

Although the colliculus is the proposed primary candidate for mediating macrovibrissal orienting (Benedetti, 1991; Hemelt and Keller, 2007; Cohen et al., 2008), little is known about the pathways that convey information from the microvibrissae once they have been contacted (Deschênes et al., 2012). Both the micro and macrovibrissae are represented extensively in the cortex, the macrovibrissae by the barrels, and the microvibrissae by a group of micro barrels, rostral to the barrel field (Benison et al., 2006; Deschênes et al., 2012). The high density of the microvibrissae and their large representation in the somatosensory cortex suggests that they do function as a high-resolution tactile sensor and supports Brecht et al.'s (1997) proposition that they function in object recognition tasks and feature detection. The orienting of the microvibrissae to objects, and their repeated touches, maximizes the number of whisker contacts with an object. In neonatal rats, this might be key to identifying textures and soft surfaces, so that they can huddle and thermoregulate effectively. In adults, the microvibrissae could aid in discriminating tactile cues such as texture and shape (Brecht et al., 1997), which may be important for adult rats to identify food items, precisely locate objects such as insect prey for biting, and to navigate effectively around their environment.

### Conflict of interest statement

The authors declare that the research was conducted in the absence of any commercial or financial relationships that could be construed as a potential conflict of interest.
